# Adverse Outcomes, Polysubstance Use, and Polypharmacy in Older Veterans

**DOI:** 10.1093/geroni/igaa057.2687

**Published:** 2020-12-16

**Authors:** Amy Byers

## Abstract

This session will provide information about adverse health outcomes, including suicide, suicide attempts and unintended death, that may be related to polysubstance use and polypharmacy in older adults, particularly older veterans. It will further provide information that will help support late-life suicide prevention and intervention efforts. Older veterans (age50 and older) have the highest number of lives lost to suicide, make up majority of the veteran population, and are highly likely to experience conditions (e.g., chronic pain, sleep disorders, musculoskeletal) associated with commonly prescribed medications that are potential markers for suicide risk (hereafter referred to as “high-risk” drug categories), including benzodiazepines, sedative-hypnotics, opioids, antidepressants, antipsychotics, and antiepileptics. The research presented in this session will highlight important patterns in high-risk drug prescribing and use and related outcomes in late life. The presentations will underscore various groups of older veterans that may be important to consider and, yet, neglected in the polysubstance and polypharmacy and suicide prevention conversation, including those who recently attempted suicide, veterans with late-life posttraumatic stress disorder (PTSD), and older veterans transitioning from prison to community. Drs. Maust and Morin will present findings on prescribing and use of high-risk medications in late life, including an overview of trends in polypharmacy and associations with suicide attempts. Drs. Byers and Barry will speak about older veterans with PTSD and those with a history of incarceration, with information on suicide, unintended death by overdose, and substance use disorder-related emergency department visits and hospitalizations, emphasizing importance of care transition models for prevention. Aging, Alcohol and Addictions Interest Group Sponsored Symposium.

